# A computationally efficient method for hand–eye calibration

**DOI:** 10.1007/s11548-017-1646-x

**Published:** 2017-07-19

**Authors:** Zhiqiang Zhang, Lin Zhang, Guang-Zhong Yang

**Affiliations:** 1 School of Electronic and Electrical Engineering, University of Leeds, Leeds, UK; 2 Hamylyn Centre, Imperial College London, London, UK

**Keywords:** Minimally invasive surgery, Robot–camera calibration, Hand–eye calibration, Optimization

## Abstract

**Purpose:**

Surgical robots with cooperative control and semiautonomous features have shown increasing clinical potential, particularly for repetitive tasks under imaging and vision guidance. Effective performance of an autonomous task requires accurate hand–eye calibration so that the transformation between the robot coordinate frame and the camera coordinates is well defined. In practice, due to changes in surgical instruments, online hand–eye calibration must be performed regularly. In order to ensure seamless execution of the surgical procedure without affecting the normal surgical workflow, it is important to derive fast and efficient hand–eye calibration methods.

**Methods:**

We present a computationally efficient iterative method for hand–eye calibration. In this method, dual quaternion is introduced to represent the rigid transformation, and a two-step iterative method is proposed to recover the real and dual parts of the dual quaternion simultaneously, and thus the estimation of rotation and translation of the transformation.

**Results:**

The proposed method was applied to determine the rigid transformation between the stereo laparoscope and the robot manipulator. Promising experimental and simulation results have shown significant convergence speed improvement to 3 iterations from larger than 30 with regard to standard optimization method, which illustrates the effectiveness and efficiency of the proposed method.

## Introduction

With increasing maturity of master–slave surgical robots, research in robotically assisted minimally invasive surgery has now focussed on the development of cooperative control and automation of certain repetitive surgical steps such as suturing, ablation, and microscopic image scanning  [1–3]. This is beneficial for the operating surgeon who can share control with the robot on low-level surgical maneuvers, freeing both perceptual and cognitive capacity on more demanding tasks that require direct human interaction [4]. Thus far, the use of vision guidance augmented with pre- and intra-operative imaging such as CT, MR and ultrasound has been applied to a range of surgical tasks including neurosurgery, orthopaedics, and cardiothoracic interventions [5–9]. However, in order to perform effective image-guided interventions, it is essential to recover the transformation between the robot coordinate frame and the endoscopic camera coordinates, which is a well-known hand–eye calibration problem in robotics.

In general, any rigid transformation can be described by two parameters: a translation vector and a rotation matrix. Thus far, a number of closed-form solutions have been proposed for hand–eye calibration. For example, Shiu and Ahmad [10] proposed to solve the robot–sensor calibration problem by estimating the orientational component and translational component separately. The orientation component was derived by utilizing the angle–axis formulation of rotation, while the translational component could be solved using standard linear system techniques once the rotational part is estimated. To simplify the process, Park and Martin [11] proposed a solution for the orientation component by taking advantage of Lie group theory to transform the orientation component into a linear system. Chou and Kamel [12] introduced quaternion to represent orientation and re-formulated the determination of the rotation matrix as a homogeneous linear least squares problem. Closed-form solution was then derived using the generalized inverse method with singular value decomposition analysis. Alternatively, Liang and Mao [13] applied the Kronecker product to get the homogeneous linear equation for the rotation matrix. Pan et al. [14] and Najifi et al. [15] also presented similar work. However, all the aforementioned methods separated the orientation component from the positional component, and errors in the rotation estimation could be propagated into the translational estimations.

Simultaneous solutions for rotational and translational components were also presented in the past decades. For instance, Lu and Chou [16] derived an eight-space formulation based on quaternion to obtain the least squares solution for the hand–eye calibration problem using the Gaussian elimination and Schur decomposition analysis. Daniilidis [17] introduced the idea of dual quaternion parameterization, which facilitated a new simultaneous solution for the hand–eye rotation and translation using singular value decomposition. Zhao and Liu [18] employed the screw motion theory to establish a hand–eye matrix equation by using quaternion, resulting in a simultaneous result for rotation and translation by solving linear equations. Andreff et al. [19] applied the Kronecker product to re-formulate the robot–sensor problem into a linear system. Least square solutions were derived to simultaneously solve the robot–sensor problem. Although all these methods can estimate rotational and translational components for the hand–eye calibration problems simultaneously, many of them involve complicated derivations.

To circumvent this problem, researchers tend to move from closed-form solutions to iterative methods due to its high efficiency and simplicity. The basic idea of iterative method is to minimize the difference between the left and right parts of the hand–eye equation or its variations. Thus far, a number of solutions have been proposed. For instance, Schmidt et al. [20] constructed a cost function using dual quaternion and quaternion multiplication properties, and classic optimization method was then applied to minimize the cost function. Strobl and Hirzinger [21] proposed a weighting schemes to construct a cost function. Similarly, they also applied classic nonlinear optimization method to solve the cost function minimization problem. Mao et al. [22] applied Kronecker product to establish a nonlinear objective function and then derived the iterative Jacobian formula to find the optimized solution using Gauss–Newton method or Levenberg–Marquet method. Zhao [23] proposed to use convex optimization, which can solve the hand–eye calibration problem in the form of a global linear optimization without starting values. Ruland et al. [24] proposed to integrate the hand–eye calibration problem into a branch-and-bound parameter space search. The presented method constituted the first guaranteed globally optimal estimator for simultaneous optimization of both components with respect to a cost function based on re-projection errors. Ackerman et al. [25] presented a unified algorithm which used gradient descent optimization on the Euclidean group. They also applied filtering to update the calibration parameters online based on new incoming data. Heller et al. [26, 27] presented several formulations of hand–eye calibration that led to multivariate polynomial optimization problems. Convex linear matrix inequality (LMI) relaxation was used to effectively solve these problems and to obtain globally optimal solutions. Wu et al. [28] and Sarrazin et al. [29] also presented similar ideas for hand–eye calibration between robotic system and vision/ultrasound systems. All these methods can solve the hand–eye calibration problem, albeit being complex to implement in practice and involving complicated calculations. It also results in low efficiency, which may be not applicable to scenarios where online hand–eye calibration must be performed regularly.

**Fig. 1:**
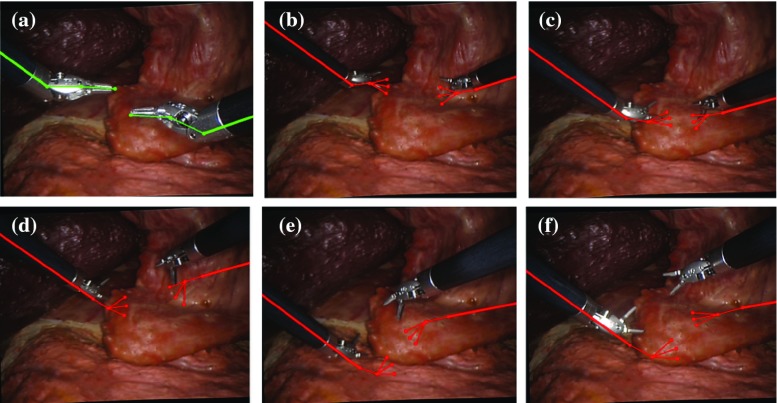
An example of re-projection of da Vinci surgical instruments by using kinematic re-projection of the model directly onto laparoscopic images. **a** The re-projection right after the calibration; **b**–**f** as time progresses and the instruments are moved away, the re-projection (shown in *red*) errors start to build up. Real-time hand–eye calibration is therefore required to re-estimate the transformation between the robot coordinate frame and the camera coordinates

In practice, the hand–eye calibration may need to be done frequently due to changes in surgical instruments and in the case of using pickup probes, their position related to the end effector is not always the same. Figure 1 shows an example of re-projection of da Vinci surgical instruments on laparoscopic images. The top left frame illustrates the re-projection right after the calibration where the error is minimal. As the instruments are moved away, the re-projection (shown in red) errors start to build up; therefore, online hand–eye calibration must be performed regularly. In order to ensure seamless execution of the surgical procedure without affecting the normal surgical workflow, it is important to derive a computationally efficient iterative method for solving hand–eye calibration. The contributions of the paper include:

- We proposed a two-step iterative method that can recover the real part and dual part of the dual quaternion efficiently and fast;
- We theoretically proved the convergence of the proposed two-step iteration method.

The proposed method was applied to determine the rigid transformation between the stereo laparoscope and the robot manipulator. Promising experimental and simulation results have shown significant convergence speeds improvement to 3 iterations from larger than 30 with regard to standard optimization method, which illustrates the effectiveness and efficiency of the proposed method.

The rest of the paper is organized as follows. The mathematical details of hand–eye calibration are given in “Problem statement” section. The proposed two-step iterative method is then introduced in “Two-step iterative method” section. Experimental results and conclusions are provided in “Experimental and simulation results and Conclusion” sections, respectively.

## Problem statement

The most common way to describe the hand–eye calibration problem is using the homogeneous transformation matrix as:

1 $$\begin{aligned} AX=XB \end{aligned}$$

where *A* and *B* are known homogeneous matrices, and *X* is the unknown transformation between the robot coordinate frame and camera coordinate frame. For each homogeneous matrix, it is in the form of

2 $$\begin{aligned} \left[ \begin{array}{cc} R &{} t \\ 0 &{} 1 \\ \end{array} \right] \end{aligned}$$

where *R* is a $3\times 3$ rotational matrix, and *t* is a $3\times 3$ translational vector. Thus, we can expand Eq. (1) as

3 $$\begin{aligned} \left[ \begin{array}{cc} R_A &{} t_A \\ 0 &{} 1 \\ \end{array} \right] \cdot \left[ \begin{array}{cc} R_X &{} t_X \\ 0 &{} 1 \\ \end{array} \right] =\left[ \begin{array}{cc} R_X &{} t_X \\ 0 &{} 1 \\ \end{array} \right] \cdot \left[ \begin{array}{cc} R_B &{} t_B \\ 0 &{} 1 \\ \end{array} \right] \end{aligned}$$

where $R_A$, $R_X$ and $R_B$ are the rotational matrix parts of *A*, *X* and *B*, and $t_A$, $t_X$ and $t_B$ are the translational parts, respectively. Equation (3) can be further simplified as:

4 $$\begin{aligned} \left[ \begin{array}{cc} R_AR_X &{} R_At_X+t_A \\ 0 &{} 1 \\ \end{array} \right] =\left[ \begin{array}{cc} R_XR_B &{} R_Xt_B+t_X \\ 0 &{} 1 \\ \end{array} \right] . \end{aligned}$$

The purpose of hand–eye calibration is to find $R_X$ and $t_X$ given *J* pair of $A_i$ and the corresponding $B_i$, where $i=1,2\ldots J$. We can have the estimation as:

5 $$\begin{aligned} \{\hat{R}_X,\hat{t}_X\}=\underset{R_X,t_x}{{\text {argmin}}}\Big \{f(R_X,t_X)\Big \} \end{aligned}$$

subject to

6 $$\begin{aligned} R_X\cdot R_X^\mathrm{T}=I \end{aligned}$$

and

7 $$\begin{aligned} {\hbox {det}}(R_X)=1 \end{aligned}$$

where *I* is the identify matrix of order 3, and ${\hbox {det}}(\cdot )$ is the determinant of a $3\times 3$ matrix. The cost function $f(R_X,t_X)$ can be defined as:

8 $$\begin{aligned} f(R_X,t_X)=\sum _{i=1}^{J}\Big \Vert A_iX-XB_i\Big \Vert \end{aligned}$$

or

9 $$\begin{aligned}&f(R_X,t_X)\nonumber \\ {}&\quad =\sum _{i=1}^{J}\Big \{\Big \Vert R_{A_i}R_{X}-R_{X}R_{B_i}\Big \Vert +\Big \Vert R_{A_i}t_{X}+t_{A_i}-R_{X}t_{B_i}-t_{X} \Big \Vert \Big \} \end{aligned}$$

where $\Vert \cdot \Vert$ is the Frobenius norm. Many algorithms, such as active set algorithm [30], interior point algorithm [31], sequential quadratic programming (SQP) algorithm [32] and so on, have been proposed so far to solve the above constrained minimization problem, but these methods tend to calculate the Jacobian matrix and Hessian matrix, which are computationally expensive. In the next section, we will present a simple two-step iterative method to solve the above constrained optimization problem.

## Two-step iterative method

In order to convert the hand–eye calibration from the homogeneous transformation matrix format into dual quaternion representation, a short introduction of quaternion is therefore given at the beginning of this section.

### Quaternion and dual quaternion

According to the Euler’s rotation theorem, any rotation can be expressed as a unit quaternion *q*. Given any two unit quaternions $p=(p_0, v_p)$ and $q=(q_0, v_q)$, the multiplication of *p* and *q* can be represented as  [33, 34]:

10 $$\begin{aligned} p\otimes q= \mathcal {R}(q)p \end{aligned}$$

or

11 $$\begin{aligned} p\otimes q= \mathcal {L}(p)q \end{aligned}$$

where $\otimes$ represents quaternion multiplication,

12 $$\begin{aligned} \mathcal {R}(q)= \left[ \begin{array}{cc} q_0 &{} \qquad -v_q^\mathrm{T} \\ v_q &{} \qquad q_0\cdot I+\lfloor v_q\times \rfloor \\ \end{array} \right] \end{aligned}$$

and

13 $$\begin{aligned} \mathcal {L}(p)= \left[ \begin{array}{cc} p_0 &{}\qquad -v_p^\mathrm{T} \\ v_p &{}\qquad p_0\cdot I-\lfloor v_p\times \rfloor \\ \end{array} \right] . \end{aligned}$$

Here, $\lfloor \times \rfloor$ is the skew-symmetric matrix/cross-product operator.

In practice, quaternion can only be used to represent orientation. In order to represent orientation and translation together, quaternion is combined with dual number theory to form dual quaternion [35]. Each dual quaternion consists of two quaternions as:

14 $$\begin{aligned} \breve{q}=q_r+q_d\varepsilon \end{aligned}$$

where $\varepsilon$ is the dual number, $q_r$ and $q_d$ are quaternion. Given any two dual quaternion $\breve{p}=p_r+p_d\varepsilon$ and $\breve{q}$, the multiplication can be defined as:

15 $$\begin{aligned} \breve{q}\otimes \breve{p}=q_r\otimes p_r +(q_r\otimes p_d+q_d\otimes p_r)\varepsilon . \end{aligned}$$

For any homogeneous transformation in the form of

16 $$\begin{aligned} \left[ \begin{array}{cc} R &{} t \\ 0 &{} 1 \\ \end{array} \right] , \end{aligned}$$

the corresponding dual quaternion representation $\breve{q}$ can be defined as:

17 $$\begin{aligned} \left\{ \begin{aligned} q_r = D2q(R) \\ q_d = \frac{1}{2}q_r\otimes t\\ \end{aligned} \right. \end{aligned}$$

where $D2q(\cdot )$ is the operator to convert a rotation matrix to the corresponding unit quaternion, and *t* is taken as a pure quaternion with 0 scale part [34, 35].

### Two-step iteration

For any i ($i=1,2\ldots J$), we have

18 $$\begin{aligned} A_iX=XB_i. \end{aligned}$$

Denote

19 $$\begin{aligned} \begin{aligned} \breve{q}_{A_i}&=q_{r,A_i}+q_{d,A_i}\varepsilon \\ \breve{q}_{B_i}&=q_{r,B_i}+q_{d,B_i}\varepsilon \\ \breve{q}_{X}&=q_{r,X}+q_{d,X}\varepsilon \\ \end{aligned} \end{aligned}$$

as the qual-quaternion representation for $A_i$, $B_i$ and *X*, respectively, Eq. (18) can thus be written as:

20 $$\begin{aligned} \breve{q}_{X}\otimes \breve{q}_{A_i}=\breve{q}_{B_i}\otimes \breve{q}_{X}. \end{aligned}$$

According to Eq. (15), the above equation can be expanded as:

21 $$\begin{aligned}&q_{r,X}\otimes q_{r,A_i} + (q_{r,X}\otimes q_{d,A_i}+q_{d,X}\otimes q_{r,A_i})\varepsilon \nonumber \\&=\,q_{r,B_i}\otimes q_{r,X} +(q_{r,B_i}\otimes q_{d,X}+q_{d,B_i}\otimes q_{r,X})\varepsilon . \end{aligned}$$

Therefore, we can have

22 $$\begin{aligned} q_{r,X}\otimes q_{r,A_i}=q_{r,B_i}\otimes q_{r,X} \end{aligned}$$

and

23 $$\begin{aligned} q_{r,X}\otimes \, q_{d,A_i}+q_{d,X}\otimes \, q_{r,A_i} =q_{r,B_i}\otimes \, q_{d,X}+q_{d,B_i}\otimes \, q_{r,X} \end{aligned}$$

According to Eqs. (10) and (11), Eq. (22) can then be written as:

24 $$\begin{aligned} \big (\mathcal {L}(q_{r,B_i}) - \mathcal {R}(q_{r,A_i})\big )q_{r,X}=0 \end{aligned}$$

and Eq. (23) can be written as:

25 $$\begin{aligned} \big (\mathcal {R}(q_{d,A_i})-\mathcal {L}(q_{d,B_i})\big )q_{r,X} = \big (\mathcal {L}(q_{r,B_i})-\mathcal {R}(q_{r,A_i})\big )q_{d,X}. \end{aligned}$$

Therefore, we can have:

26 $$\begin{aligned} H_{l,i}q_{r,X}=H_{r,i}q_{d,X} \end{aligned}$$

where

27 $$\begin{aligned} H_{l,i}=\left[ \begin{array}{c} \mathcal {L}(q_{r,B_i}) - \mathcal {R}(q_{r,A_i}) \\ \mathcal {R}(q_{d,A_i})-\mathcal {L}(q_{d,B_i}) \\ \end{array} \right] \end{aligned}$$

and

28 $$\begin{aligned} H_{r,i}=\left[ \begin{array}{c} 0_{4\times 4} \\ \mathcal {L}(q_{r,B_i})-\mathcal {R}(q_{r,A_i}) \\ \end{array} \right] . \end{aligned}$$

Define

29 $$\begin{aligned} H_l=\left[ \begin{array}{c} H_{l,1} \\ H_{l,2} \\ \vdots \\ H_{l,J} \\ \end{array} \right] \end{aligned}$$

and

30 $$\begin{aligned} H_r=\left[ \begin{array}{c} H_{r,1} \\ H_{r,2} \\ \vdots \\ H_{r,J} \\ \end{array} \right] \end{aligned}$$

we can have

31 $$\begin{aligned} H_{l}\cdot q_{r,X}=H_{r}\cdot q_{d,X} \end{aligned}$$

and given any initial value for $q_{r,X}$ as $q_{r,X}^0$, $q_{r,X}$ and $q_{d,X}$ can be estimated as:

1. Set index $$n=1$$;
2. Calculate $$q_{d,X}^n$$ as:
  32 $$\begin{aligned} q_{d,X}^n= H_{r}^{+}\cdot H_{l}\cdot q_{r,X}^{n-1} \end{aligned}$$
  where $(\cdot )^{+}$ is the pseudo-inverse operator.
3. Calculate $$q_{r,X}^{n}$$ as
  33 $$\begin{aligned} q_{r,X}^{n}=H_l^{+}\cdot H_{r}\cdot q_{d,X}^n. \end{aligned}$$
4. Set $n=n+1$ and repeat steps $2-4$ until $q_{r,X}^n$ and $q_{d,X}^n$ converge.
5. Re-scale the magnitudes and set the $q_{r,X}$ and $q_{d,X}$ estimates as
  34 $$\begin{aligned} \begin{aligned} {\hat{q}_{r,X}}&=\frac{q_{r,X}^n}{\Vert q_{r,X}^n\Vert } \\ {\hat{q}_{d,X}}&=\frac{q_{r,X}^n}{\Vert q_{r,X}^n\Vert }q_{d,X}^n. \\ \end{aligned} \end{aligned}$$
6. The estimation of *X* can thus be calculated as:
  35 $$\begin{aligned} \hat{X}=\left[ \begin{array}{cc} q2D(\hat{q}_{r,X}) &{} 2\cdot (\hat{q}_{r,X})^*\otimes \hat{q}_{d,X}\\ 0 &{} 1 \\ \end{array} \right] \end{aligned}$$
  where $q2D(\cdot )$ is the operator to convert a unit quaternion to the corresponding rotation matrix and $^*$ represents the quaternion conjugate. To make the dimension of *X* correct, only the vector part of quaternion $2\cdot (\hat{q}_{r,X})^*\otimes \hat{q}_{d,X}$ is selected in the above equation [34].

#### Theorem 1

The $q_{r,X}^n$ and $q_{d,X}^n$ can always converge to obtain the ground truth for $q_{r,X}$ and $q_{d,X}$ via the two-step iteration methods.

#### Proof

Similar to Eq. (5), the purpose of the two-step iteration is to minimize

36 $$\begin{aligned} \Big \Vert H_{l}\cdot q_{r,X}-H_{r}\cdot q_{d,X}\Big \Vert \end{aligned}$$

which means that $q_{r,X}^n$ and $q_{d,X}^n$ can converge to obtain the ground truth for $q_{r,X}$ and $q_{d,X}$ only if:

37 $$\begin{aligned} \begin{aligned} \Big \Vert H_{l}\cdot q_{r,X}^n-&H_{r}\cdot q_{d,X}^{n+1}\Big \Vert \leqslant \Big \Vert H_{l}\cdot q_{r,X}^n-H_{r}\cdot q_{d,X}^{n}\Big \Vert \end{aligned} \end{aligned}$$

and

38 $$\begin{aligned} \begin{aligned} \Big \Vert H_{l}\cdot q_{r,X}^n-&H_{r}\cdot q_{d,X}^{n}\Big \Vert \leqslant \Big \Vert H_{l}\cdot q_{r,X}^{n-1}-H_{r}\cdot q_{d,X}^{n}\Big \Vert . \end{aligned} \end{aligned}$$

For Eq. (37), we can have

39 $$\begin{aligned}&\Big \Vert H_{l}\cdot q_{r,X}^n-H_{r}\cdot q_{d,X}^{n+1}\Big \Vert \nonumber \\&=\Big \Vert H_{l}\cdot q_{r,X}^n-H_{r}\cdot H_{r}^{+}\cdot H_{l}\cdot q_{r,X}^{n}\Big \Vert \nonumber \\&=\Big \Vert (I- H_{r}\cdot H_{r}^{+})\cdot H_{l}\cdot q_{r,X}^n\Big \Vert \end{aligned}$$

**Fig. 2:**
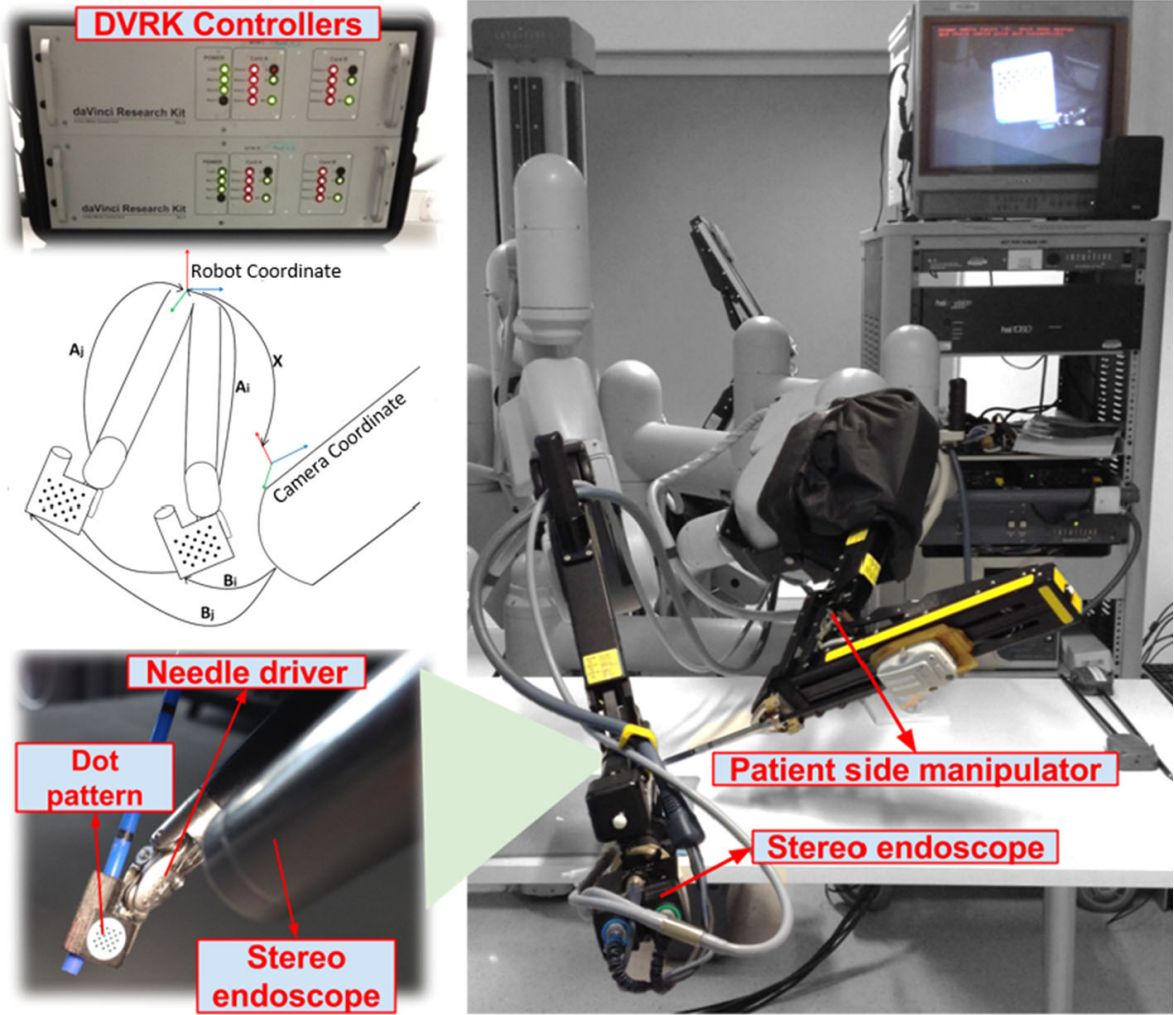
The hand–eye data were collected using the da Vinci Research Kit (DVRK): the pose of the tool in the robot base coordinate was obtained via the robot forward kinematics, while the corresponding pattern pose in the camera coordinate was derived using a pair of stereo images containing the pattern

For any matrices $\varUpsilon$ and $\varPsi$, $\Vert I-\varUpsilon ^{+}\varUpsilon \Vert <\Vert I-\varPsi ^{+}\varUpsilon \Vert$ is always satisfied unless $\varUpsilon =\varPsi$ [36], so

40 $$\begin{aligned}&\Big \Vert H_{l}\cdot q_{r,X}^n-H_{r}\cdot q_{d,X}^{n+1}\Big \Vert \nonumber \\&\leqslant \Big \Vert \Big (I- H_{r}\cdot H_{r}^{+} \cdot H_{l}\cdot q_{r,X}^{n-1}\ (H_{l}\cdot q_{r,X}^n)^+\Big ) H_{l}\cdot q_{r,X}^n\Big \Vert \nonumber \\&=\,\Big \Vert H_{l}\cdot q_{r,X}^n-H_{r}\cdot H_{r}^{+} \cdot H_{l}\cdot q_{r,X}^{n-1}\Big \Vert \nonumber \\&=\,\Big \Vert H_{l}\cdot q_{r,X}^n-H_{r}\cdot q_{d,X}^{n}\Big \Vert . \end{aligned}$$

For Eq. (38), we can also have

41 $$\begin{aligned}&\Big \Vert H_{l}\cdot q_{r,X}^n-H_{r}\cdot q_{d,X}^{n}\Big \Vert \nonumber \\&=\,\Big \Vert H_{l}\cdot H_l^{+}\cdot H_{r}\cdot q_{d,X}^n-H_{r}\cdot q_{d,X}^{n}\Big \Vert \nonumber \\&=\,\Big \Vert (H_{l}\cdot H_l^{+}-I)H_{r}\cdot q_{d,X}^{n}\Big \Vert \end{aligned}$$

Similar to Eq. (40), we can have

42 $$\begin{aligned}&\Big \Vert H_{l}\cdot q_{r,X}^n-H_{r}\cdot q_{d,X}^{n}\Big \Vert \nonumber \\&\leqslant \Big \Vert \Big (H_{l}\cdot H_l^{+}\cdot H_{r}\cdot q_{d,X}^{n-1}\nonumber \\&\qquad \qquad \quad \cdot (H_{r}\cdot q_{d,X}^{n})^+-I\Big )H_{r}\cdot q_{d,X}^{n}\Big \Vert \nonumber \\&=\,\Big \Vert H_{l}\cdot H_l^{+}\cdot H_{r}\cdot q_{d,X}^{n-1}-H_{r}\cdot q_{d,X}^{n}\Big \Vert \nonumber \\&=\,\Big \Vert H_{l}\cdot q_{r,X}^{n-1}-H_{r}\cdot q_{d,X}^{n}\Big \Vert \end{aligned}$$

$$\square $$

## Experimental and simulation results

In order to evaluate the performance of the proposed hand–eye calibration algorithm, detailed simulation and laboratory experiments were carried out. The simulation study was based on the Monte Carlo method, which was carried out on a workstation with 3.40 GHz Intel Core i7 processor and 16G RAM. For the experimental results presented in this paper, as shown in Fig. 2, the hand–eye data were collected using the da Vinci Research Kit (DVRK) which included mechanical components from the first-generation da Vinci robotic surgical system [37] and controllers/software APIs developed by Johns Hopkins University [38]. A stereo laparoscope held by a robotic arm was used to capture various poses of a calibration pattern that contains 21 key dots. The pattern is grasped by a surgical needle driver tool that is controlled by a patient side manipulator (PSM). During the experiment, the pattern was moved to different position and orientation of the robot’s workspace. The pose of the tool in the robot base coordinate was obtained via the robot forward kinematics, while a corresponding pattern pose in the camera coordinate was derived using a pair of stereo images containing the pattern.

### Simulation study

In practical experiments, it is difficult to know the ground truth of the rigid transformation between camera coordinate system and robot coordinate system; therefore, we resort to simulation study with known parameters. In this simulation, the rotation part of a rigid transformation *X* was randomly set by the *ZYX* convention Euler angles as roll ${-}$0.7309 rad, pitch 0.0513 rad and yaw ${-}$2.0804 rad, while the translational vector was set as $[0.7822,~0.1513,~-0.4811]^\mathrm{T}$ (unit meter). Thus, the random rigid transformation *X* can be written as:

$$\begin{aligned} X=\left[ \begin{array}{cccc} 0.7436 &{} -0.6667 &{} -0.0513 &{} 0.7822\\ -0.3590 &{} -0.3333 &{} -0.8718 &{} 0.1513\\ 0.5641 &{} 0.6667 &{} -0.4872 &{} -0.4811\\ 0 &{} 0 &{} 0 &{} 1\\ \end{array} \right] . \end{aligned}$$

and 5 pairs of $A_i$ and the corresponding $B_i$ were then generated based on the *X* within a space of $0.25\mathrm{m}\times 0.25\mathrm{m}\times 0.25\mathrm{m}$.

To simulate the orientation estimation error in the $A_i$, a random selected $3\times 1$ vector $\delta v_{i}$ with less than 0.035 magnitude [(to make sure the rotation angle is less than our empirical value of 0.0349 rad (2$^\circ$)] was applied to generate a small rotational error matrix $\delta R_{i}$ for each $R_{A_i}$ as:

$$\begin{aligned} \delta R_{i}= \lfloor \delta v_{i}\times \rfloor +I \end{aligned}$$

Thus, the $R_{A_i}$ used in our simulation is

$$\begin{aligned} R_{A_i}=\delta R_{i}R_{A_i}. \end{aligned}$$

Similarly, a small rotation error was also added to $R_{B_i}$ using the same method. To simulate the translation error as well, a zero mean noise with standard deviation 0.002 m was added to both $t_{A_i}$ and $t_{B_i}$.

**Fig. 3:**
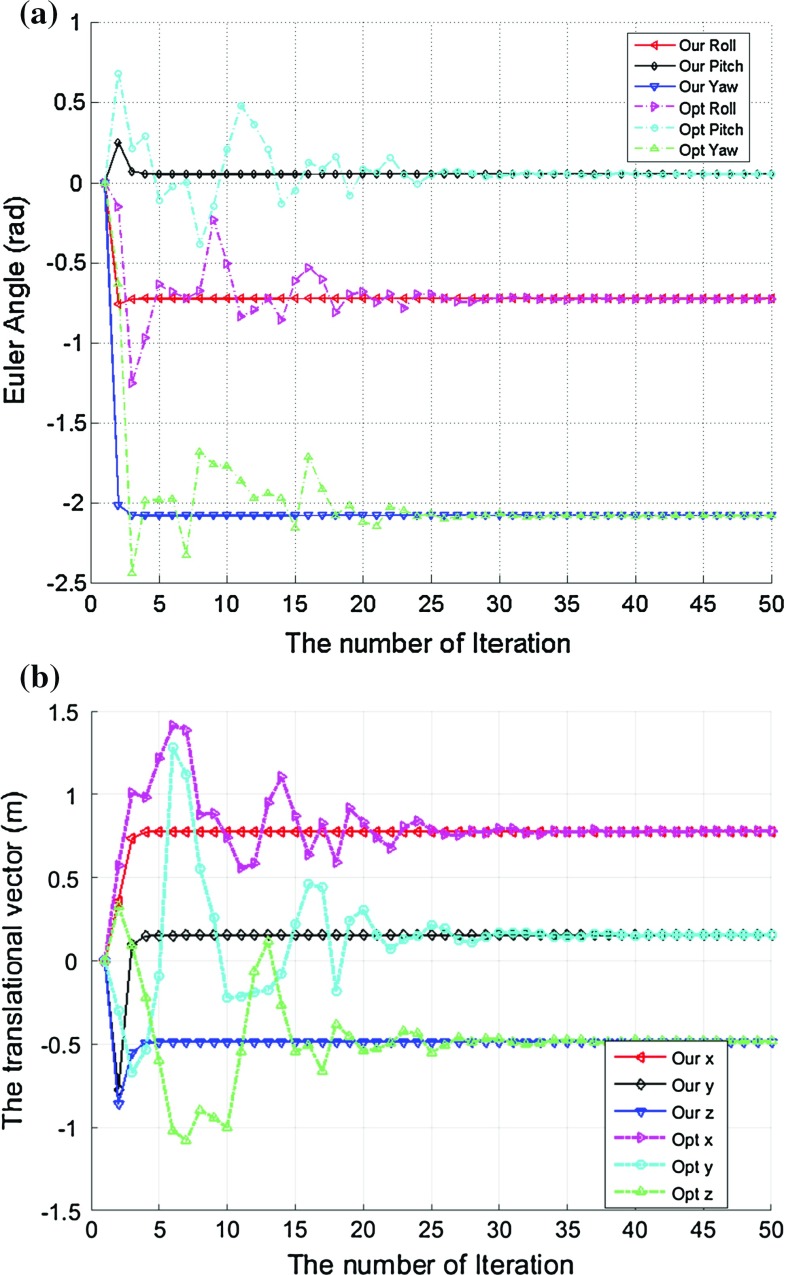
The iterative results for the rigid transformation matrix *X* estimation. **a** The rotational part given by the Euler angles, **b** the translational vector estimation results. It is very clear that the proposed two-step iteration method can converge much faster than the traditional optimization method

**Fig. 4:**
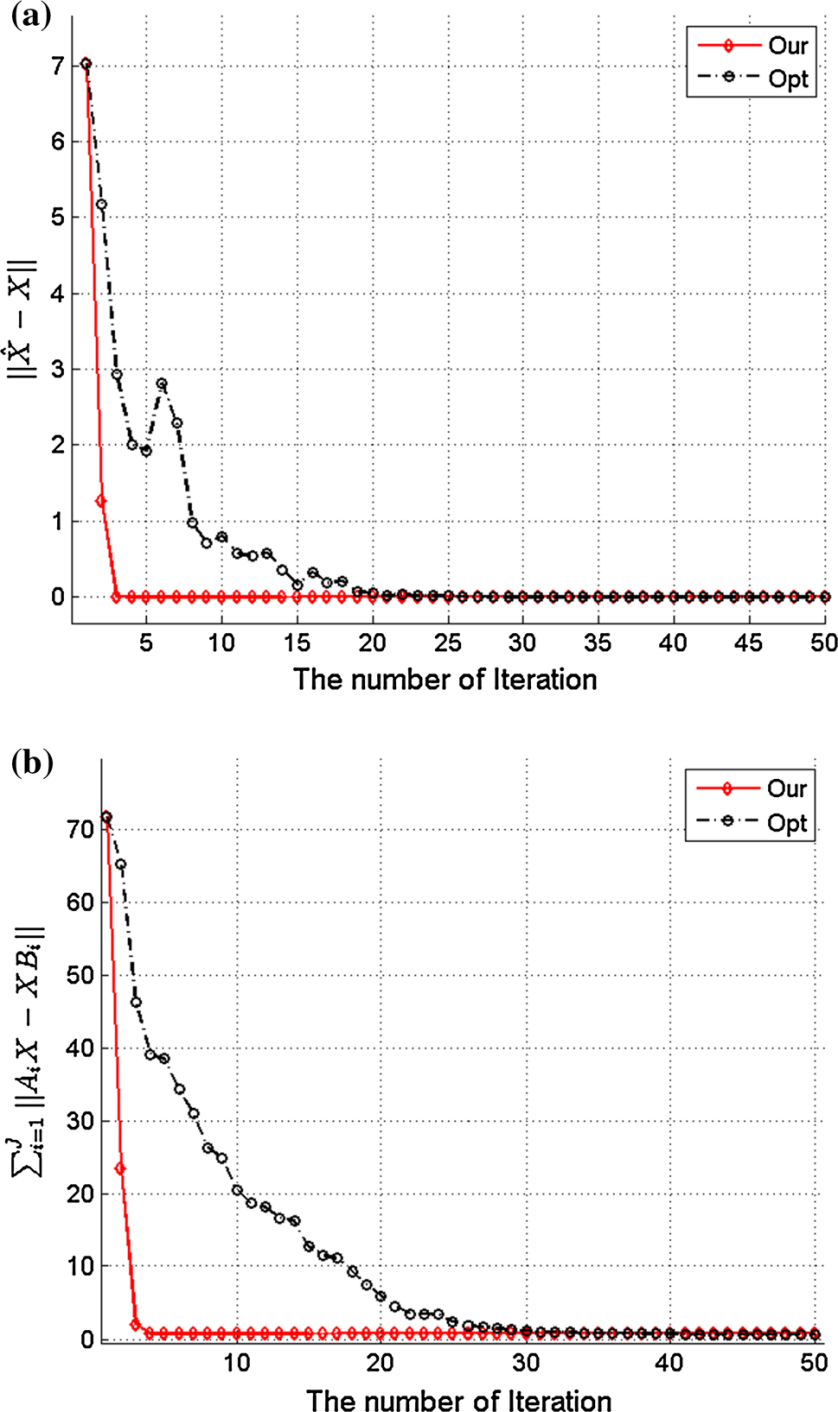
The estimation error for rigid transformation *X*. **a** The error between the *X* estimation and its ground truth, **b** the value of the cost function. It is very clear that the proposed two-step iteration method can converge much faster than the traditional optimization method

Figure 3 shows the iterative results for the estimation of the rigid transformation *X*: Fig. 3a gives the rotational results given by the *ZYX* Euler angles, while Fig. 3b provides the translation vector estimation results. For systematic comparison, we also implemented the SQP algorithm to optimize the constrained problem in Eq. (5), and the results derived from the SQP algorithm are also shown in Fig. 3. As we can see from the figure, both methods can generate the $R_X$ and $t_X$ estimations accurately, but the proposed method is much faster than the traditional optimization method. After 3 iterations using our method, the estimation for *X* is almost equal to its ground truth value. Although the optimization method can also converge to the ground truth of *X*, convergence speed is much slower and it needs more than 30 iterations to achieve the same accuracy.

To better illustrate of estimation error, Fig. 4a presents the error between the *X* estimation and its ground truth, while Fig. 4 shows the value of the cost function $\sum _{j=1}^{J}\Big \Vert A_iX-XB_i\Big \Vert$. From the figures, it is obvious that either the estimation error or the cost function will decrease to 0 after only 3 iterations using our method. To achieve the same accuracy, the traditional optimization method needs at least 30 iterations. Furthermore, the convergence process of our method to find *X* is much smoother. After every single iteration, the estimation of *X* will get closer to the ground truth and the value of the cost function will get smaller. In contrast, the estimation of *X* using the SQP method may divert from the ground truth although the value of the cost function gets smaller after some certain iterations. It has been noted that the SQP optimization method took about 2 s to complete all the iterations, while our method only took less than 0.1 s in our simulation by using the same computing hardware. In fact, the SQP algorithm usually requires to calculate the value of cost function more than 10 times within an iteration, and it also involves sophisticated Hessian and Jacobian matrix operations, which are computationally expensive. However, our proposed method only requires some basic matrix operations, such as multiplication and inverse, which therefore make our method much more efficient than the traditional optimization method. This is attractive for direct implementation of the algorithm on embedded system as part of the robotic hardware.

For this paper, the simulation was repeated for 500 times using different pairs of *A* and *B* (different rotation, translational, and perturbations), and statistical results for *X* estimation are given in Table 1. It is obvious that the proposed two-step iterative method converges after 3 iterations with negligible errors, while the traditional optimization based methods needs more than iterations. In conclusion, the above analysis has shown that the proposed two-step iteration method can solve the hand–eye calibration problem accurately and efficiently.

We also noticed that when we increased rotation angle error from 0.0175 to 0.175 rad and translation error from 0.001 to 0.01 m for any $A_i$ and corresponding $B_i$, both estimations of *X* from our method and traditional optimization start to deviate from the ground truth. The main reason is the accuracy of *X* estimation depends on the qualities of *A* and *B*. The larger the errors in *A* and *B* are, the more inaccurate the *X* estimation is.

**Table 1:** Iterative results over 500 simulations (shown as mean ± STD)

	$$\big \Vert X-\hat{X}\big \Vert $$	
	Our	Optimization
Iteration 2	2.2330 ± 9.1455	5.5626 ± 0.6195
Iteration 5	0.0003 ± 0.0004	1.9939 ± 0.6316
Iteration 10	0.0002 ± 0.0001	1.0979 ± 0.5014
Iteration 20	0.0002 ± 0.0001	0.0550 ± 0.0358
Iteration 40	0.0002 ± 0.0001	0.0004 ± 0.0002
Iteration 50	0.0002 ± 0.0001	0.0002 ± 0.0001

### DVRK experiments

**Fig. 5:**
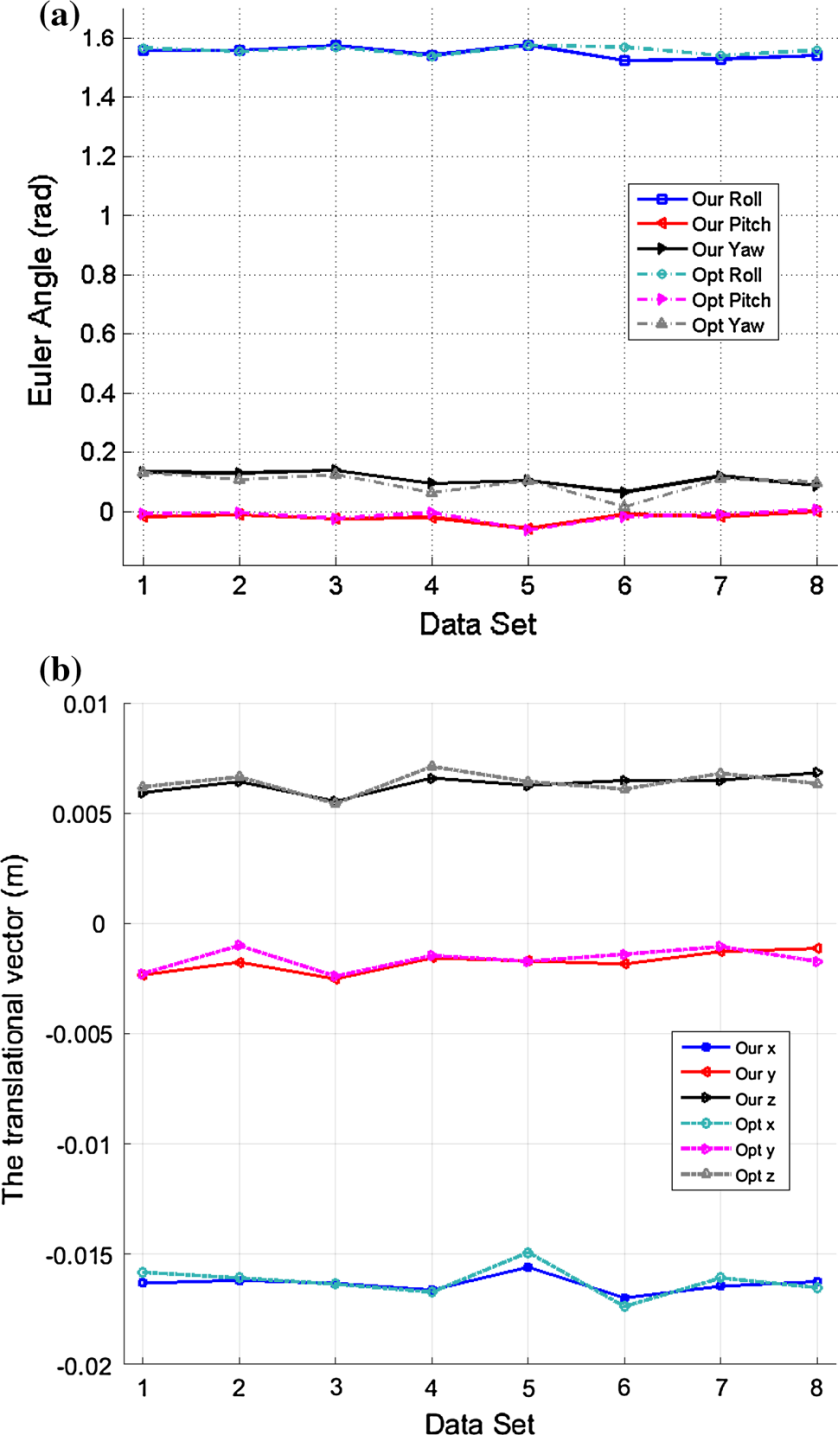
The rigid transformation estimation results using the DVRK: **a** the rotation estimation results, **b** the translation estimation results. During the experiments, the same calibration method was repeated 8 times using different data sets. Although there is no ground truth for the rigid transformation, the estimation results have shown good consistency, which illustrates the robustness of our proposed method

The proposed two-step iteration method was then applied to estimating the rigid transformation between the stereo laparoscope and the robot manipulator, as shown in Fig. 2. During the experiment, the robot manipulator was randomly moved to different positions and orientations within the robot work space, and the pose information was then extracted from the robot forward kinematics. Meanwhile, the pose information in the camera coordinate was also derived from the stereo images containing the 21 dots pattern. Eight data sets have been acquired, and in each data set, the robot manipulator was randomly placed at 5 different orientations and positions to generate 5 pairs of *A* and *B*. Figure 5 shows the hand–eye calibration results based on the 8 independent data sets. As we can see from the figure, either the Euler angles or the translational vector estimation results are similar with very small deviation throughout all the trials performed, and the deviations are also very small. The consistency among all the 8 trials indicates the good repeatability of the proposed method. Similarly, the traditional optimization method can also get the comparable results, except it is more computationally expensive. It is also worth mentioning that although there is no ground truth for the rigid transformation between the stereo laparoscope and the robot manipulator, the consistency of the data illustrates the robustness and reproducibility of our proposed method.

**Fig. 6:**
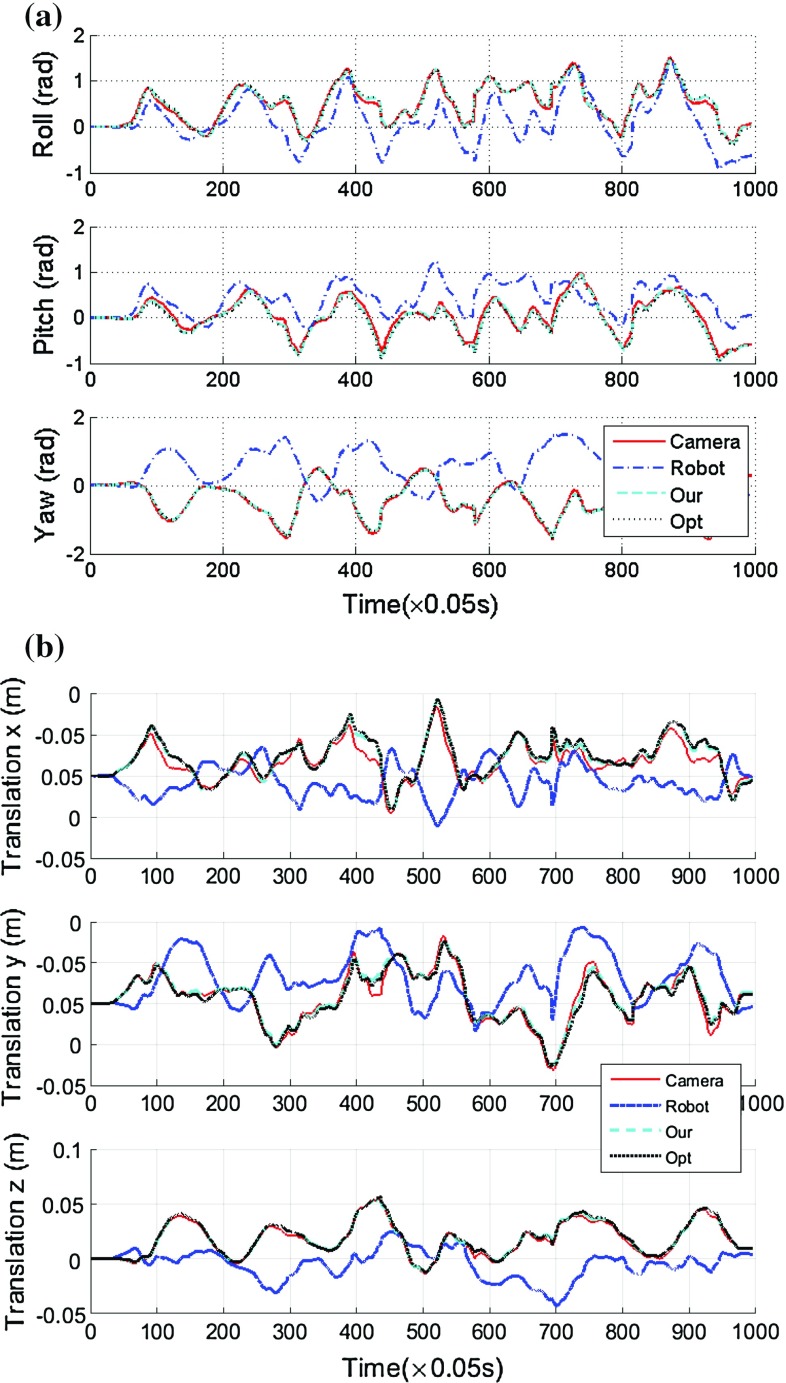
The projection of poses in the camera coordinate system into the robot system. **a** The projection results for the rotational part, **b** the projection results of the translational part. The projection was completed using the *X* estimated from our two-step iteration method and the traditional optimization method, respectively

After applying the proposed two-step iterative method to determine the rigid transformation between the stereo laparoscope and the robot manipulator, we then transformed the poses in the robot coordinate system into the camera system. In this experiment, we moved the robot manipulator to different positions and rotations. Similar to the last experiment, the continuous pose information in the robot frame was acquired from the robot forward kinematics, while the corresponding pose information in the camera coordinate was derived from the stereo images. We then converted the poses in the robot coordinate system into the camera system using the estimated *X*, and compared the differences. Figure 6 shows the transformation results: the red solid line indicates the poses extracted from the stereo images directly, the blue dash-dotted line indicates the poses derived from the robot forward kinematics, the cyan dashed line represents the convention using the *X* estimated by the proposed method, while the black dotted line marks the transformation using the *X* derived from the traditional optimization method.

**Fig. 7:**
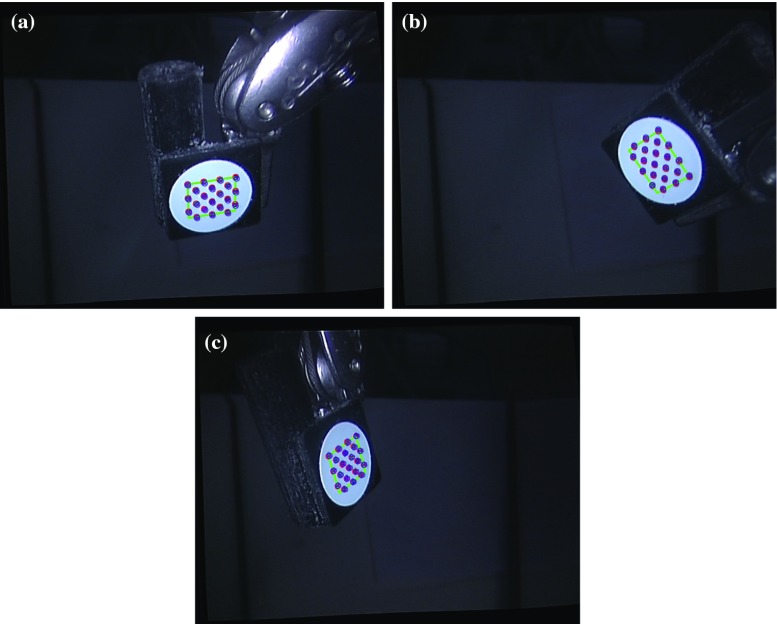
The projection of robot homogeneous transformation matrix to the image space for randomly selected three frames: 147, 374 and 885. The *blue crosses* indicate the projection, while the *red ones* represent the dots detected in the image space directly for homogeneous transformation matrix derivation in the camera space. It shows that the errors between the projection and direct detection are very small

As shown in Fig. 6, either the Euler angles or the translation vector can have significant differences for the poses given in the robot coordinate system and the camera system; therefore, hand–eye calibration must be completed to find the rigid transformation between them in advance. It is also clear that the proposed two-step iterative method can determine the rigid transformation between the stereo laparoscope and the robot manipulator and project pose information in the robot coordinate system into the camera system accurately. We also noticed that although the convergence speeds of optimization based methods are much slower than our proposed iterative method, they can also provide accurate sensor conversion. To better illustrate the advantage of the proposed two-step iteration method to solve the hand–eye calibration problem, the quantitative comparison results of the projection are also provided, as shown in Table 2. The projection using the *X* estimated by the proposed method is slightly better than the transformation using the *X* derived from the traditional optimization method. In general, our method has smaller RMS error and better correlation. In order to visualize the errors of the hand–eye calibration using the proposed method, Fig. 7 shows he projection of robot homogeneous transformation matrix to the image space for randomly selected three frames: 147, 374 and 885. The blue crosses indicate the projection,while the red ones represent true dot locations. It can be seen that the re-projection errors are very small. In summary, the above analyses illustrate the fact that the proposed two-step iteration method can solve the hand–eye calibration problem and determine the rigid transformation between the stereo laparoscope and the robot manipulator accurately and efficiently.

**Table 2:** The RMS, mean, SD and correlation coefficients of the projection of robot homogeneous transformation matrix to the image space

	Optimization calibration		Our calibration	
	RMS (Mean, SD)	Correlation coefficient	RMS (Mean, SD)	Correlation coefficient
Roll	0.0226	0.9885	0.0176	0.9914
(Unit rad)	($-$0.0198 ± 0.0686)		($-$0.0073 ± 0.0550)	
Pitch	0.0323	0.9861	0.0231	0.9902
(Unit rad)	(0.0795 ± 0.0641)		(0.0463 ± 0.0564)	
Yaw	0.0195	0.9948	0.0182	0.9952
(Unit rad)	($-$0.0196 ± 0.0583)		($-$0.0131 ± 0.0560)	
Translation *x*	1.2161	0.9550	0.9663	0.9663
(Unit mm)	($-$2.3400 ± 3.0459)		($-$1.6659 ± 2.5573)	
Translation *y*	0.7859	0.9804	0.7773	0.9821
(Unit mm)	(0.3183 ± 2.4621)		($-$0.7218 ± 2.3468)	
Translation *y*	0.6594	0.9960	0.5828	0.9956
(Unit mm)	($-$1.5204 ± 1.4232)		($-$1.1235 ± 1.4580)	

### Continuous tracking experiment

**Fig. 8:**
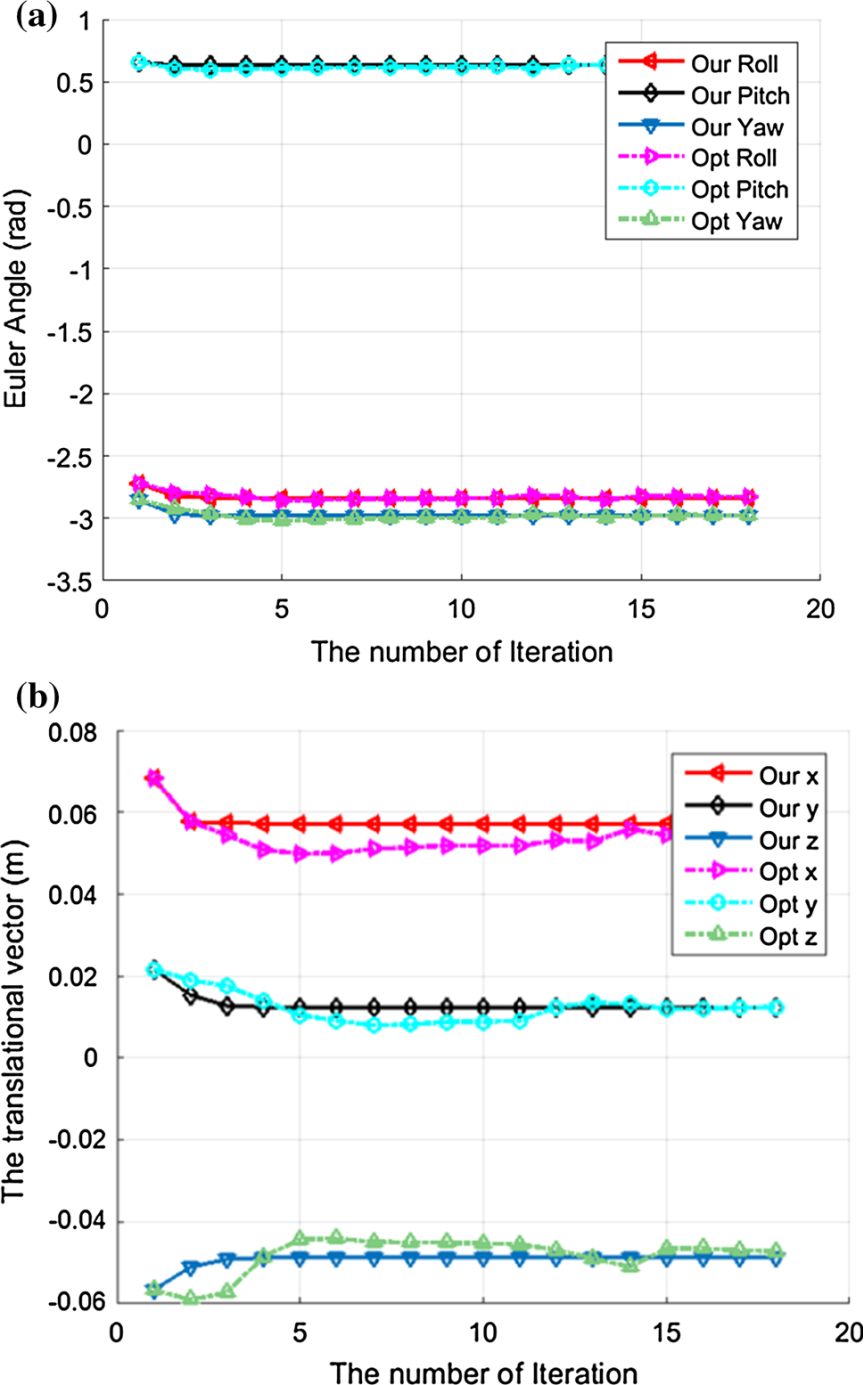
The iterative results for the re-calibration after the laparoscope was shifted for a couple of millimeters. **a** The rotational part given by the Euler angles, **b** the translational vector estimation results. It is very clear that the proposed two-step iteration method can converge much faster than the traditional optimization method

One of the main advantages of the proposed two-step iteration method is its high efficiency and fast convergence speed, which is crucial for online hand–eye calibration. During a robot-assisted surgery, the stereo laparoscope can be moved for better field of view and visualization. This will invalidate the hand–eye calibration, and an in-vivo re-calibration procedure is required without affecting a surgeon’s workflow. To simulate this scenario, the laparoscope was moved for a couple of millimeters to introduce small change in calibration parameter *X* after the robot and laparoscope was registered via the hand–eye calibration in our last experiment. The previous calibration result was used as the initial value to re-perform the hand–eye calibration. Figure 8 shows the re-calibration results using two sets of new poses, and the proposed two-step iterative method only needs two to three steps to converge, while the traditional optimization method requires almost 15 steps, particularly for the translational part to converge.

**Fig. 9:**
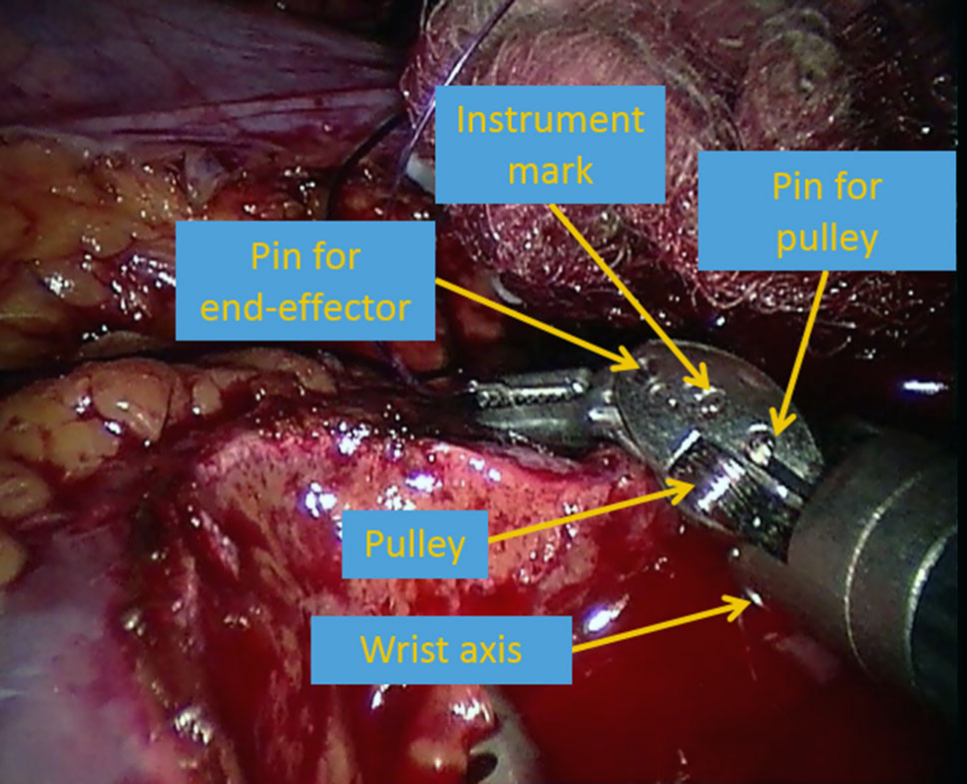
The illustration of feature points on a instrument that can be applied extract the new pairs of *A* and *B*

It should be noted that key dot calibration pattern was used to extract the new poses in the camera coordinate system, which may only be applicable to limited number of surgical applications. To apply the proposed method on more general surgical tasks, markerless tracking techniques, such as instrument feature points tracking [39, 40] as shown in Fig. 9, are needed. Although Pachtrachai et al. [41] had successfully applied robotic surgical instruments with well-known geometry as the calibration object for hand–eye calibration, how to accurately and robustly extract the new poses based on the feature point in the presence of occlusion and stained instrument is yet to be solved. We will also evaluate the benefits of fast online calibration in robot-assisted surgery when we have positive progress in surgical instruments feature points tracking.

## Conclusion

In conclusion, we have presented a computationally efficient method to solve the hand–eye calibration equation $AX=XB$ given several pairs of rigid transformations *A* and the corresponding *B*. In our method, dual quaternion was introduced to represent the rigid transformation, and a two-step iterative method was then proposed to recover the real part and dual part of the dual quaternion simultaneously, which could thus be applied to estimate rotation and translation for the rigid transform. The proposed method was applied to determine the rigid transformation between the stereo laparoscope and the robot manipulator. Promising experimental and simulation results were achieved to illustrate the effectiveness and efficiency of the proposed method.

Our future work will investigate how to accurately and robustly extract the new poses based on the feature point in the presence of occlusion and stained instrument, and apply the proposed method in vivo.
